# The Key Role of Purine Metabolism in the Folate-Dependent Phenotype of Autism Spectrum Disorders: An In Silico Analysis

**DOI:** 10.3390/metabo10050184

**Published:** 2020-05-06

**Authors:** Jan Geryk, Daniel Krsička, Markéta Vlčková, Markéta Havlovicová, Milan Macek, Radka Kremlíková Pourová

**Affiliations:** Department of Biology and Medical Genetics, 2nd Faculty of Medicine, Charles University and University Hospital Motol, V Úvalu 84, 150 06 Prague, Czech Republic; dkrsicka@gmail.com (D.K.); marketa.vlckova@lfmotol.cuni.cz (M.V.); marketa.havlovicova@fnmotol.cz (M.H.); milan.macek.jr@lfmotol.cuni.cz (M.M.J.); radka.pourova@fnmotol.cz (R.K.P.)

**Keywords:** autism, ASD, folate, blocked metabolite, purine, *ADSL*, *GART*, *PFAS*, *PPAT*, *PAICS*, *ATIC*, cerebral folate deficiency, Flux Balance Analysis (FBA), metabolic modeling

## Abstract

Folate deficiency in the critical developmental period has been repeatedly associated with an increased risk of Autism spectrum disorders (ASD), but the key pathophysiological mechanism has not yet been identified. In this work, we focused on identifying genes whose defect has similar consequences to folate depletion in the metabolic network. Within the Flux Balance Analysis (FBA) framework, we developed a method of blocked metabolites that allowed us to define the metabolic consequences of various gene defects and folate depletion. We identified six genes (*GART*, *PFAS*, *PPAT*, *PAICS*, *ATIC*, and *ADSL*) whose blocking results in nearly the same effect in the metabolic network as folate depletion. All of these genes form the purine biosynthetic pathway. We found that, just like folate depletion, the blockade of any of the six genes mentioned above results in a blockage of purine metabolism. We hypothesize that this can lead to decreased adenosine triphosphate (ATP) and subsequently, an S-adenosyl methionine (SAM) pool in neurons in the case of rapid cell division. Based on our results, we consider the methylation defect to be a potential cause of ASD, due to the depletion of purine, and consequently S-adenosyl methionine (SAM), biosynthesis.

## 1. Introduction

Autism spectrum disorders (ASD) are neurodevelopmental disorders characterized by impaired social communication and a tendency to exhibit stereotypical behavior. The prevalence of ASD ranges from 1.2% to 1.7%, depending on the type of study and population analyzed [1,2,3,4].

ASD represents a serious socio-economic issue, since the lifelong direct and indirect costs for one person suffering from ASD exceeded 2.3 million USD in 2014 [5]. Thus far, the only known effective therapy is an evidence-based behavioral intervention [6,7,8]. The etiology of ASD is both heterogeneous and complex, and continues to be intensively studied. A number of different genetic syndromes, genomic variants, and recently, multiple environmental and metabolic disturbances, have been associated with ASD [9,10,11,12] so far.

Folate depletion is a well-known factor for an increased risk of neural tube defects [13,14]. Recently, several neuropsychiatric disorders, such as ASD or schizophrenia, have been associated with defective folic acid metabolism [15]. The disruption of folate metabolism, irrespective of genetic factors [16,17], encompasses malnutrition [18], insufficient folate absorption due to microbiome imbalance [19], and a blockade of folate receptors on the blood–brain barrier or placental barrier by Folate Receptor Auto Antibodies (FRAA) [20,21]. Therefore, folates can be present in the body, but their absorption, interconversion, or transport can be disrupted in many ways. By local organ-specific blocking of folate transport, FRAA can cause significant changes in physiological folate levels and lead to Cerebral Folate Deficiency Syndrome (CFD) [20]. The compensation of FRAA-based CFD has resulted in improvements in ASD symptoms [22].

**Figure 1:**
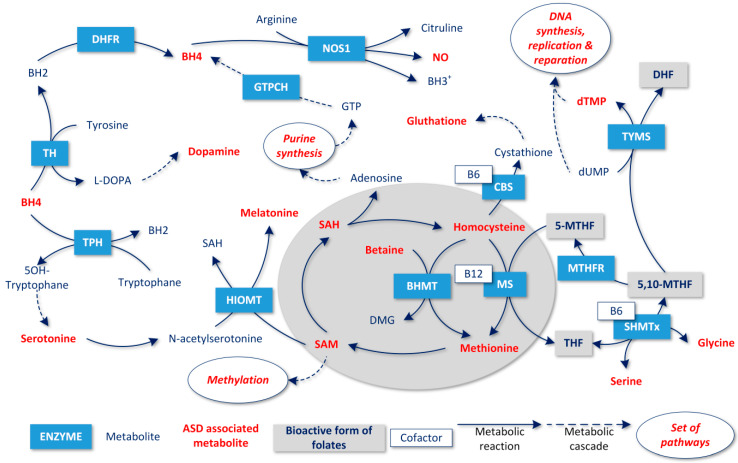
Involvement of folate metabolites associated with Autism spectrum disorders (ASD) (in red).

Folates are essential substances in humans, and are necessary for the biosynthesis of nucleotides and neurotransmitters and for methylation pathways influencing more than 100 metabolic reactions. The most important folate-dependent pathways relevant to a potential association with ASD are shown in Figure 1 and are as follows:

- The production of methionine from homocysteine, followed by its conversion to S-adenosylmethionine (SAM), which primarily provides methyl groups for DNA, RNA, chromatin, protein, and phospholipid methylation, in order to maintain the physiological regulation of gene expression during brain development and maturation. Altered DNA methylation has been connected to ASD [23], as well as mutations in subunits of DNA methyltransferase (*DNMT1*, *DNMT3A*, *DNMT3B*, and *DNMT3*) [24,25,26];
- Homocysteine degradation to cystathionine and consecutive glutathione synthesis (transsulfuration pathway) produces protective factors against reactive oxygen species (ROS). A reduction in the methionine regeneration cycle also results in a reduction in antioxidant synthesis activity [27]. In the case of folate depletion, methionine can be regenerated by an alternative pathway that converts choline to betaine and then to dimethylglycine via betaine homocysteine methyltransferase (BHMT) [28]. Lowered levels of betaine have been found in ASD [29];
- The production of tetrahydrobiopterin (BH4) via the synthesis of purines is a substrate for the precursors of catecholamines. Hyperserotonemia has been associated with the induction of ASD in animal models [30]; conversely, melatonin levels decrease, and its compensation has been clinically studied in ASD [31]. BH4 also enables the synthesis of l-3,4-dihydroxyphenylalanine (L-DOPA) and a recent study showed abnormal levels of dopamine in patients with ASD [32]. The synthesis of purines utilizes adenosine, which is a byproduct of the methionine regeneration cycle [33];
- BH4 is also necessary for the synthesis of nitric oxide (NO). Under healthy conditions, BH4 is regenerated, but if the concentration of BH4 is lowered, peroxynitrite (ONOOH) is produced. Its accumulation leads to the hyperexcitation of NMDA receptors, and together with the accumulation of homocysteine, induces the apoptosis of neurons [15]. Oxidized pterins inhibit NO synthase and further lower the synthesis of NO [33];
- The utilization of purines in the activation and suppression of the Cell Danger Response (CDR) influences the vital state of the cell via changes in mitochondrial metabolism. Changes in purinergic signaling have also been associated with ASD [34,35,36].
- Synthesis of pyrimidine as a substrate for DNA synthesis and replication.

The role of folate metabolism in relation to ASD was clearly demonstrated by a recent study of Howsmon at al. [37].

The authors of this study demonstrated that on the basis of blood levels of metabolites directly related to folate-dependent one-carbon metabolism (FOCM) and transsulfuration pathways (TS), it is possible to discriminate ASD patients from age-matched healthy controls with a high accuracy. The authors also showed that the severity of ASD measured by the ‘Vineland Adaptive Behavior Composite Score’ can be predicted based on the same metabolite set. Follow-up studies confirmed and extended these findings [38].

It was also shown in another study that pregnant mothers already having an ASD child exhibited an altered FOCM and TS metabolite profile in their anamnesis in comparison to pregnant mothers without an ASD child [39].

The study of Vargason et al. provided evidence that treatment decreasing the difference in FOCM and TS metabolite levels between ASD patients and healthy controls can improve ASD-related symptoms [40].

Based on the aforementioned studies, Vargason et al. developed a causative metabolic model of the methionine cycle and TS pathway, in order to find critical parameters determining the steady state of FOCM and TS metabolites [41]. They deduced the parameters of the model from a set of experimentally measured blood levels of FOCM and TS metabolites from ASD patients and healthy controls. The authors observed that only a subset of parameters reflect the apparently different FOCM and TS metabolite profiles between ASD patients and healthy controls. Following the application of sensitivity analysis, the rate of glutamylcysteine synthesis was identified as an important factor governing the steady state of FOCM and TS metabolites.

The work of Vargason et al. represents an important step towards finding a mechanistic explanation for the altered metabolite profile in ASD patients and potentially suggests novel therapeutic targets. Besides, it should be noted that their metabolic model encompasses only a small subset of human cell metabolism and that the concentrations of FOCM and TS metabolites can also be significantly affected by more distant processes within a complex metabolic network. Therefore, based on this concept, we decided to analyze the association of FOCM with ASD within genome-scale human metabolic networks.

Genome-scale metabolic networks are mostly studied from the viewpoint of the network topology and flux balance analysis (FBA), since most of the parameters needed to study whole network dynamics are generally missing [42]. FBA is a constraint-based method for the prediction of metabolic fluxes within a metabolic network, which assumes a constant concentration of all metabolites [43,44,45]. The best approximation of this constant concentration model is quickly dividing cells within a constant environment. Despite such a limitation, the FBA-based approach provides important insights into the metabolism of different species, including humans [46,47]. Gene deletions or defects are modeled by setting the upper and lower boundaries of gene-associated reactions to zero or reducing them to a narrow interval. Flux variability analysis (FVA) is performed after this modification to identify the metabolic consequences of the deletion. The result of FVA is a set of reactions that are blocked or limited due to the deletion, suggesting which metabolic systems will be affected by the deletion [48,49]. We used an analogical approach in our study and focused on the loss of ability to synthesize metabolites de-novo after gene knockout.

We applied the concept of blocked metabolites to the problem of folate deficiency (see Methods). We used this approach to investigate the metabolic consequences of intracellular folate depletion, in order to search for genes whose knockout has similar metabolic consequences to intracellular folate depletion. By modeling folate deficiency at an intracellular level, we represent the common expected consequence of several pathological conditions, such as FRAA positivity, defects in folate transporters, or simple malnutrition, which are all associated with ASD. When discussing folate deficiency in the following text, we consider intracellular folate deficiency.

Our goals were (1) to find potential gene defects with functionally similar consequences to folate deficiency, (2) to identify new mechanisms or candidate genes that might be associated with ASD, and (3) to contribute to the elucidation of folate deficiency pathophysiology. Similar, but less sophisticated analyses, were performed in our previous work, where associations between folate depletion and known ASD-associated metabolic genes were investigated [50]. The known ASD-associated genes (i.e., *TMLHE*, *ADSL*, *CBS*, and *AMPD1*) have been found to be directly associated with reactions dependent on folate [50]. Here, we apply the method of blocked metabolites developed within the FBA framework, in order to identify and compare exclusively metabolically essential relationships. This was not permitted by the methodology of our previous study. Consequently, we did not have to deal with the problem of a combinatorial explosion in identifying folate/gene-dependent metabolites. The presented method allowed us to stratify the results from our previous work and, as a result, to more fully investigate the consequences of metabolic defects that can connect folate deficiency with known inborn or postnatally acquired errors of metabolism related to ASD.

## 2. Results

### 2.1. Blocked Metabolites Associated with Gene Knockouts

We constrained the Recon 2.2 model to uptake only compounds contained within the RPMI_1640 medium (see Appendix A). We then computed blocked metabolites and blocked reactions for every gene knockout in Recon 2.2 formed by the union of all human metabolic paths (“supercell”) [51]. Most gene knockouts (≈78%) did not produce any blocked metabolites. Regarding blocked reactions, ≈57% of genes had no blocked reactions after knockout. This finding reflects the inherent robust nature of metabolic networks, as noted in other studies [52], as well as the fact that the investigated network is a “super-network” composed of virtually all tissue-specific metabolic networks in the human body. We observed that 376 genes out of a total of 1675 genes in Recon 2.2 had a non-empty blocked metabolite set (22.5%).

### 2.2. Systemic Folate Deficiency from the Viewpoint of Blocked Metabolites

We simulated systemic folate deficiency as an absence of all intracellular forms of folate in Recon 2.2. We deleted all reactions that consumed any intracellular folate metabolite, in order to find additional metabolites blocked as a consequence of folate deficiency (see Methods). Every blocked metabolite identified in this way was dependent on folate in the sense that the blocked metabolite could not be synthesized without the presence of a folate pool within the cell. We hypothesized that, in-vivo, any disturbance in folate levels can also disturb the levels of dependent blocked metabolites, which we had identified.

As a consequence of deleting all of the reactions consuming folate metabolites, we identified 760 blocked metabolites within the Recon 2.2 reconstruction. This set of blocked metabolites was then compared with a set of blocked metabolites associated with knockouts of each of the 1675 metabolic genes in the Recon 2.2 model. The Jaccard coefficient ($J_{C}$) was computed for every comparison in these result sets (see Table S1 in supplementary materials).

**Figure 2:**
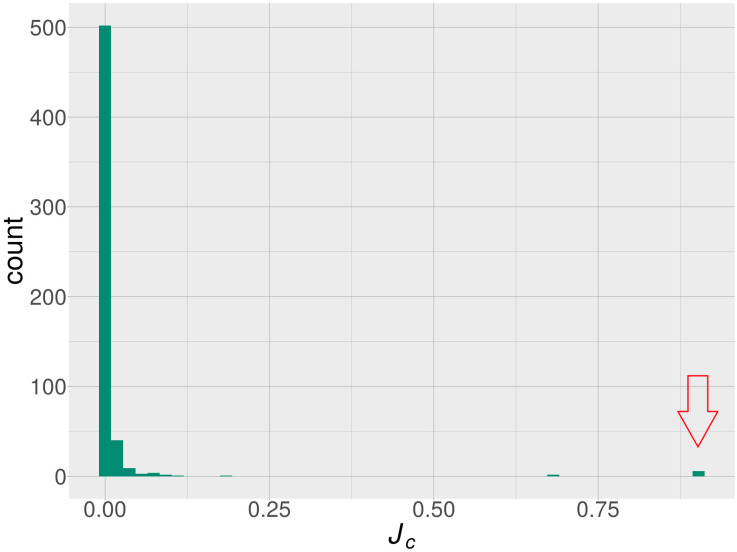
Distribution of Jaccard coefficient ($J_{C}$) values measuring the similarity between folate deficiency and individual genes with a ‘non-empty’ blocked metabolite set within the Recon 2.2 model. The cluster of six purine-metabolism genes mentioned in the main text is indicated by an arrow.

In our model, based on Recon 2.2, two important metabolites—BH4 and SAM—were blocked in simulated folate deficiency. We found only six genes (*GART*, *PFAS*, *PPAT*, *PAICS*, *ATIC*, and *ADSL*) that had BH4 and SAM in their blocked metabolite sets (see Table 1). These six genes also exhibited a relatively high overlap of their blocked metabolite sets with the blocked metabolite set of folate deficiency ($J_{C}\in 〈0.896,0.903〉$). The aforementioned genes formed a separate cluster which significantly deviated from the expected value in the distribution plot of $J_{C}$ values (see Figure 2). We found three genes (*GCH1*, *PTS*, and *SPR*) with BH4, but not SAM, in their blocked metabolite sets. We did not find any genes that had SAM, but did not have BH4, in their result sets of blocked metabolites.

### 2.3. Cerebral Folate Deficiency from the Viewpoint of Blocked Metabolites

Applying the same methodology to the metabolic model of cerebral cortex neurons produced by the CORDA method, we obtained very similar results to those produced in the previous case of systemic folate deficiency. We identified 83 blocked metabolites resulting from the elimination of all reactions consuming folate metabolites. Subsequently, we found the same six genes as in the previous case, which had a high overlap with folate deficiency in terms of blocked metabolites (see Table 1). The only difference was that the de-novo synthesis of BH4 was blocked in the native state of the metabolic model of cerebral cortex neurons.

### 2.4. Implications for the Pathophysiology of ASD

The genes whose sets of blocked metabolites had the most significant overlap with blocked metabolites in simulated folate deficiency were found to be the same in both metabolic reconstructions studied (systemic and cerebral cortex). These genes (*GART*, *PFAS*, *PPAT*, *PAICS*, *ATIC*, and *ADSL*) are shown in Table 1, together with the degree of overlap with the modeled folate deficiency in terms of blocked metabolites. Both the Jaccard coefficient and the generalized Jaccard coefficient exhibited a high degree of similarity between the blocked metabolite sets associated with the above-mentioned six genes and the blocked metabolite set associated with the modeled folate deficiency (Table 1). 

**Figure 3:**
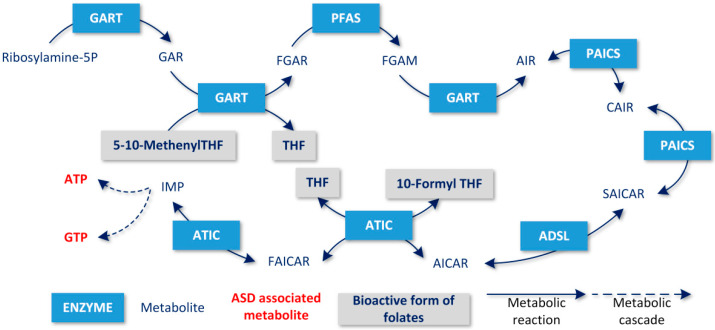
Result set of genes forming a comprehensive part of the de-novo synthesis of purines.

All of the above-identified six gene products formed the purine biosynthetic pathway (see Figure 3). Among them, only two genes (ADSL and *ATIC*) were associated with neurodevelopmental disorders.

*ADSL* gene mutations cause a disorder associated with the ASD phenotype, called adenylosuccinate lyase (ADSL) deficiency (OMIM #103050). ADSL deficiency has traditionally been categorized into three forms. The first form is lethal shortly after birth; the second form results in severe psychomotor retardation, an early onset of seizures, and microcephaly; and the third form exhibits mild to moderate psychomotor retardation, with seizures presenting later in development and/or several cognitive impairments, such as ASD [53].

*ATIC* gene mutations have been associated with AICAR transformylase/IMP cyclohydrolase deficiency (OMIM # 608688) manifesting with severe neurodevelopmental disorder (comprising intellectual disability, hypotonia, and seizures), dysmorphic features, and congenital blindness, due to optical atrophy, but this phenotype is only based on a single case [54]. Due to the devastating neurological picture and relatively low age of the 4-year-old patient, the possibility of the presence or later development of ASD symptoms could not be excluded in connection with *ATIC* mutations. The findings in the patient also resemble the symptoms of the second form of ADSL deficiency.

The fact that folate deficiency and ADSL (and possibly also ATIC) deficiency both result in symptoms similar to ASD and, at the same time, exhibit very similar blocked metabolite sets, suggests a possible common pathophysiological mechanism that could lead to the development of ASD. In our model, the knockout of any gene presented in Table 1 resulted in blocked purine biosynthesis, which led to blocked de-novo synthesis of BH4, since the product of purine biosynthesis, guanosine triphosphate (GTP), is a BH4 precursor (see Figure 1). Similarly, adenosine triphosphate (ATP) is an SAM precursor, which explains why SAM production is blocked as a result of blocked purine biosynthesis.

The modeled folate deficiency resulted in a block of purine biosynthesis, because two steps of the purine biosynthetic pathway (catalyzed by the GART and ATIC enzymes) require a folate derivative as a cofactor (see Figure 2). Therefore, blocked purine biosynthesis is common in the modeled folate deficiency and knockouts of each of the six above-mentioned genes. The blocking of purine biosynthesis is also able to block de-novo BH4 and SAM production.

To test whether purine biosynthesis blocking is the only mechanism explaining how modeled folate deficiency causes blocked SAM production, we removed folate dependency from the two previously mentioned steps of the purine biosynthesis pathway in the metabolic reconstruction. The *GART*- and *ATIC*-encoded enzymes or reactions were also removed from the set of reactions consuming folate which were used in the folate deficiency model. Then, we ran a new search for folate deficiency-associated blocked metabolites using this slight modification of the metabolic network. We found that SAM production was restored by removing folate dependency from the purine biosynthesis pathway in situations of folate depletion. This result clearly shows that purine blocking is the only pathway by which folate deficiency blocks SAM production within the presented model.

We found three genes (*GCH1*, *PTS*, and *SPR)* whose knockout resulted in a block of BH4, but not SAM, i.e., purine biosynthesis remained functional in this case. These genes exhibit very little overlap with folate deficiency in terms of blocked metabolites and are clinically associated with a more severe neurological disability in which ASD symptoms are virtually impossible to assess [55,56,57].

## 3. Discussion

Based on our results, we postulated the hypothesis that the ATP pool is reduced, since the neurons divide during a critical developmental period and cannot be fully replenished due to ADSL deficiency or folate deficiency. This decrease in the ATP pool is followed by a reduction in the SAM pool, which is critical for DNA methylation. There is increasing evidence that methylation impairment plays an important role in the development of ASD [58,59]. Our hypothesis is consistent with the hypothesis of purine deficiency, which states that the pathogenesis of ADSL deficiency is caused by the depletion of purines during embryonic and early development. ATP depletion can occur when the rate of proliferation activity is so high that ADSL is unable to replenish purines at the required rate. In later phases of development, cell division rates decrease, and ADSL activity becomes able to maintain purine levels. This could explain the experimental findings in which patients with *ADSL* deficiency had normal purine levels in many tissues in the postnatal period [60].

The important blocked metabolites (ATP and SAM) related to methylation have a non-zero maximum turnover rate in both simulated systemic and cerebral deficiencies, and are thus not blocked from the viewpoint of the conventional definition [61]. This is generally true for all metabolites that can be regenerated in a cycle. The deficiency of the de-novo synthesis of such metabolites would thus be increasingly manifested during periods of rapid cell division, such as brain development and maturation. Without functional de-novo synthesis, the metabolite pool would then be reduced exponentially with the increasing number of cell divisions.

The experimental work of Maddocks et al. offers support for our hypothesis [62]. The authors investigated the effect of the amino acid serine on DNA methylation using two cancer cell lines. Unexpectedly, they found that serine supports DNA methylation by de-novo ATP synthesis. They showed that serine starvation significantly decreases the transfer of methyl groups from methionine to DNA compared to a serine-supplemented culture. They demonstrated that serine starvation significantly decreases the levels of ATP, SAM, and S-adenosylhomocysteine (SAH), and that carbon from supplemented serine was propagated to ATP, SAM, and SAH. They also showed that conditions inhibiting purine biosynthesis could radically decrease the amount of ATP and SAM, as well as methyl group transfer from methionine to DNA. These results suggest a high degree of sensitivity of the DNA methylation process to the size of the ATP pool, which must be replenished by de-novo synthesis after cell division.

In addition to its common role in oxidative phosphorylation (OXPHOS) processes, ATP fulfills another important signaling function in the activation of the Cell Danger Response (CDR), which fundamentally disrupts the primary function of a certain number of mitochondria. OXPHOS is inhibited, and mitochondria produce ROS and other metabolites, providing adequate healing or apoptosis processes. After the source of danger subsides, the cell returns to its original state. Cells with a significant portion of their mitochondria activated (CDR) reduce their growth, division, maturation, and proliferation. The transition between the activation and inhibition of CDRs is subject to purinergic signaling, in which ATP plays an important role [34]. Under such conditions, disruption of the folate supply may be a significant factor in CDR activation and the consequent disruption of neuronal division, proliferation, and maturation. A longer CDR induction process during critical developmental periods may affect the development of ASD.

Conversely, the results of our study found that the blockade of ATP synthesis, caused by disruption of the folate supply or genes encoding enzymes in the purine biosynthetic pathway, was a possible cause of ASD development. However, a broader context has to be taken into account for interpretation—a number of biochemical processes described as part of the CDR (SAM regeneration, ATP synthesis, and adenosine synthesis) are directly dependent on folate delivery and metabolism [34] (see Figure 1). Therefore, it can be assumed that purine metabolism plays a key role in the pathophysiology of the ASD phenotype, both at metabolic and signaling levels.

We also found that BH4 de-novo synthesis is blocked in both the *ADSL* knockout and folate deficiency model. It is known that BH4 is essential for the biosynthesis of several neurotransmitters and its depletion could potentially also play an important role in ASD development.

The incompleteness of the metabolic network reconstructions used in the present work could have influenced our results. We observed that the metabolic reconstruction of Recon 2.2 has 1631 (≈31%) blocked metabolites in its native (unchanged) state. The metabolic reconstruction of the cerebral cortex neuron has 386 blocked metabolites (≈12%) in its native state. Additionally, the selected growth medium can influence the blocked metabolite set of a metabolic network. We observed that the number of blocked metabolites increased by 1094 after limiting the Recon 2.2 model to growth on RPMI_1640 medium and increased by 1083 after limiting the cortex neuron metabolic model to growth on the same medium in our model [55].

A third important aspect involves the tissue specificity of the metabolic reconstruction because, in the pathophysiology of ASD, we were mostly focused on the CNS. Therefore, in addition to the native Recon 2.2 model, we performed an analysis of the cerebral cortex neuron model with the goal of approximating, as far as possible, the metabolism of neurons during development. Many genes, silenced in adult tissues, are active during developmental periods. Therefore, the cerebral cortex neuron metabolic model may not exactly correspond to the metabolic network, which is active in neurons during early brain development.

Despite the general limitations of the present study, we can conclude that our proposed mechanism of SAM depletion (i.e., caused by ATP depletion due to ADSL deficiency or folate deficiency) is robust with respect to the above-mentioned issues. The adenosine molecule is a critically important metabolite for every human cell, and only one biosynthetic pathway for adenosine is known. This corresponds to the fact that purine biosynthetic pathways must be active in every dividing cell. Similarly, SAM is an important donor of methyl groups for DNA methylation reactions in humans. It is highly unlikely that any further computational reconstruction of embryonic neuronal metabolism will render these pathways inactive. Similarly, any growth medium that does not support the de-novo biosynthesis of purines in dividing cells is not appropriate.

Another important limitation of our study involves the method used to model the consequences of gene knockouts. In our approach, a blocked metabolite was defined as one which could not be synthesized after gene knockout (i.e., their maximal de-novo production rate is zero after gene knockout). This implies that no alternative pathway from the precursors in the growth medium to the metabolite exists after knockout. We hypothesize that the essentiality of a gene product for the biosynthesis of a particular metabolite increases the chance for the metabolite to be affected by various genetic defects in the gene. However, it is also possible that a metabolite will be affected by the knockout of a gene, which is not essential for its biosynthesis or does not reduce its maximal production rate in FBA analysis. These (non-essential) associations cannot be detected by our method. A broader analysis including these (non-essential) associations could be performed using a simple network topological method at the expense of a reduced specificity of the results. One of the possible ways to find all potential associations, including non-essential ones, was presented in our previous work [50]. However, the task of finding metabolites that cannot be de-novo synthesized after gene knockout is much harder using the network topological approach. The FBA framework allowed us to perform this task very simply. This work can also be viewed as stratification of our previous results on more or less probable gene candidates associated with ASD. From this perspective, the more probable candidate genes are those identified using the blocked metabolite approach (i.e., *GART*, *PFAS*, *PPAT*, *PAICS*, *ADSL,* and *ATIC*). We found that gene defects with non-empty blocked metabolite sets (type 1 metabolites (see Methods)) have a greater chance of being detrimental to the cell, because alternative pathways leading to the blocked metabolites, which could compensate for the defect, do not exist. We tested this hypothesis and found that metabolic disease genes are significantly enriched within the set of genes with a non-empty blocked metabolite set. However, despite this result, more than 69% of metabolic disease genes have empty blocked metabolite sets. It remains to be seen if this discrepancy can be reduced by using appropriate tissue-specific metabolic reconstructions and growth media or explained by the higher severity of disorders associated with genes having non-empty blocked metabolite sets.

It is also known that the pathological effect of certain inherited metabolic diseases is caused by metabolite accumulation due to defective enzymes that cannot process the metabolite at the appropriate rate. Blocked metabolite sets are not relevant for the pathophysiology of these diseases.

We are aware of the general limitations of the applied methodology. Real metabolic processes exhibit a highly dynamic nature which cannot be captured by the flux balance model. The dynamic aspects can play an important role in the pathogenesis of ASD and other related diseases. Currently, it is not possible to construct a precise dynamic model of cellular metabolism, since the majority of kinetic constants are missing. Despite this fact, we believe that the coarse-grained approach used within the manuscript can also produce relevant hypotheses for future investigations.

The etiology of ASD is diverse and comprises multiple genetic and environmental factors. Here, we only focused on one possible mechanism and demonstrated that purine deficiency represents a common pathogenic link for specific environmental (i.e., folate deficiency-related) and genetic (*ADLS*) factors.

## 4. Materials and Methods

### 4.1. Flux Balance Analysis Terminology

Flux balance analysis (FBA) is a constraint-based method for analyzing metabolic networks. FBA is typically used to predict the reaction rates (fluxes) within a metabolic network in a steady state. The steady-state assumption is formulated as S_V_ = 0, where S denotes a stoichiometric matrix with dimensions m × n, where m is the number of metabolites and n is the number of reactions within the metabolic network. Additionally, v denotes the vector of reaction fluxes. The formula states that net concentration changes of all metabolites within the network are zero. Other constraints used in FBA are the lower and upper bounds of reaction fluxes formulated as lb < v < ub. These constraints are used in order to define the growth medium for a virtual cell by manipulating the lb and ub of selected exchange reactions.

The typical task for FBA is to find values of v in such a way that biomass production is maximized. This task is solvable using linear optimization and, in summary, can be formulated as follows: maximize c^T^v, subject to Sv = 0 and lb < v < ub, where c denotes the vector indicating the degree to which individual reactions contribute to the production of biomass. The same linear program can be used to maximize or minimize an arbitrary set of reactions contained within the metabolic network by a suitable choice of vector c. To test whether the de-novo synthesis of arbitrary metabolite is blocked, we maximized one reaction representing the sink of that metabolite. Therefore, the vector c was zero, except for one element corresponding to the sink reaction of the investigated metabolite (see the next chapter for a more extensive explanation).

Blocked metabolites are conventionally defined as a metabolite with a maximum flux-sum equal to zero or smaller than some reliable limit when practical. The flux sum (Φ) is defined as follows:

(1) $$\Phi =0.5\sum_{j}\left|S_{i,j}v_{i}\right|,$$

where *S_i,j_* is the stoichiometric coefficient of reaction *j*, and *v_j_* is the reaction flux of the same reaction [61].

### 4.2. Blocked Reactions and Metabolites

Metabolic function can be viewed as the production of a specific metabolite or a group of metabolites. It seems natural to define the functional consequence of a metabolic defect as a set of metabolites whose production will decrease due to the defect.

We can distinguish two types of metabolites that are affected by gene knockout: Type 1 metabolites that cannot be de-novo synthesized in any amount (blocked metabolites), and Type 2 metabolites that can be de-novo synthesized, but whose maximal production rate is decreased with respect to the state before gene knockout. Type 1 metabolites imply that all biosynthetic paths leading from the input metabolite set to a type 1 metabolite are blocked by gene knockout. Type 2 metabolites imply the existence of an alternative unaltered pathway or pathways leading from some subset of the input metabolites contained within the growth medium to type 2 metabolites. Because there are no alternative biosynthetic pathways that can compensate for the defect, we hypothesized that type 1 metabolites are altered by the gene defect with a higher probability than type 2 metabolites under real conditions. We focused on type 1 metabolites in this paper.

In order to define blocked metabolites in terms of FBA, we added an irreversible sink reaction for every metabolite within the FBA model. A “sink reaction” is defined as a reaction that consumes a metabolite, but does not generate any product. Sink reactions are used in situations where the production rate of certain metabolite(s) needs to be measured, for example, when measuring the rate of biomass formation. A positive flux in the sink reaction that consumes the metabolite implies that the metabolite can be continuously bio-synthetized within the cell from precursors from the growth media.

We defined “blocked metabolites” in the FBA model as a set of metabolites where the maximal flux of their sink reactions equals zero.

We defined blocked metabolites associated with gene knockout as the set theoretical difference between blocked metabolites before and after gene knockout. We observed that a substantial number of metabolites were blocked in the native state of the model due to constraints imposed by the growth medium.

We then defined blocked reactions analogously to the above definition, i.e., a set of reactions whose maximum absolute flux is zero. We also defined blocked reactions associated with gene knockout as a set theoretical difference between blocked reactions after knockout and blocked reactions before knockout.

We did not use the definition of blocked metabolites based on the flux-sum [61], because metabolites within cycles that cannot be synthesized de-novo are not blocked following this definition.

The maximum flux-sum will be nonzero in situations where the metabolite is regenerated within a cycle, but cannot be synthesized de-novo. If we use our definition, such a metabolite will be blocked. This difference can be biologically important, especially in situations involving rapid cell division, such as brain growth and early maturation. The pools of cofactors like folate are reduced during cell division, and the regeneration of these pools to their original size requires de-novo synthesis or external influx.

By our definition of blocked metabolites, we were able to detect metabolites working in cycles that have blocked de-novo synthesis.

We modeled folate deficiency by setting the lower and upper bounds of all reactions consuming any form of folate to zero.

A more straightforward option for modeling folate deficiency can be realized by blocking exchange reactions that import folate into the cell. However, the folate cycle would still remain active in this situation, and the folate turnover flux could still achieve the same flux magnitudes as when importing folate was allowed. A metabolite whose de-novo synthesis only requires folate as a cofactor will not be blocked in this situation, because folate is regenerated within the cycle. We cannot see where a folate deficit would be propagated in this situation, which is why we modeled folate deficiency by blocking all reactions consuming any form of folate.

The program code used for the identification of blocked reactions and metabolites associated with gene knockouts and for the pairwise comparison of blocked sets of reactions/metabolites is available at https://github.com/geryk/Blocked-metabolites-reactions-analysis. The algorithm was developed in Matlab as an extension of the Cobra Toolbox [63].

### 4.3. Measurements of Blocked Metabolite Set Overlap

To compute the degree of overlap between two sets of metabolites, we used the Jaccard coefficient (*J_C_*). It is defined as the intersection size divided by the union size of the two sets. Formally,

(2) $$J_{C}\left(A,B\right)=\frac{\left|A\cap B\right|}{\left|A\cup B\right|},$$

where *A* and *B* are the two sets of metabolites.

The generalized Jaccard coefficient is defined as follows:

(3) $$J_{Cg}\left(x,y\right)=\frac{\sum_{i}\min\left(x_{i},y_{i}\right)}{\sum_{i}\max\left(x_{i},y_{i}\right)},$$

where *x_i_* is a proportional reduction of the maximal production rate of metabolite *i* after condition **x** is applied to the native metabolic network. Analogically, *y_i_* is a proportional reduction of the maximal production rate of metabolite *i* after condition **y** is applied to the native metabolic network. i∈(1,…,n), where *n* is the number of metabolites in the metabolic network.

## 5. Conclusions

We used the concept of blocked metabolites to analyze cerebral folate deficiency in the pathogenesis of ASD. We found a high similarity between folate deficiency and ADSL deficiency in terms of blocked metabolites. A closer look at the overlapping metabolites of these simulated deficiencies suggests that ATP depletion may play a significant role in the development of ASD. This result would not be achievable using the conventional definition of blocked metabolites, which is based on metabolite turnover rates.

According to our results, we hypothesize that in both ADSL and folate deficiencies, the ATP pool is not properly replenished, as new neurons are created during mitosis in the early phases of brain development, which can lead to the decreased methylation of DNA in newly created neurons.

The ADSL gene was found, as in our previous work, to be associated with a reaction that was indirectly dependent on folate [64]. Our study confirmed this finding using an entirely different approach and proposes a more detailed mechanism to explain how purine deficiency, caused by ADSL or folate deficiency, can lead to the depletion of ATP and subsequently to the impairment of DNA methylation during cell division. If the supply of folates is disrupted during critical developmental phases of rapid cell division, growth, and maturation of the CNS, for example, based on FRAA positivity in the prenatal period or in CFD based on antibodies postnatally and in early childhood, it could have serious consequences for ATP synthesis and cell signaling and significantly contribute to ASD development. This convincing theoretical observation requires more clinical research in this area to explore clinical consequences and the exact contribution that these findings can make to medical care.

## Figures and Tables

**Table 1:** Six genes exhibiting the highest similarity between their blocked metabolite sets and the blocked metabolite set associated with folate deficiency. $J_{C}$, Jaccard coefficient; $J_{Cg}$, generalized Jaccard coefficient (for definition see methods).

Gene Name	Supercell (All Cells)		Cerebral Cortex Neuron Only	
	$J_{C}$	$J_{Cg}$	$J_{C}$	$J_{Cg}$
GART	0.9762	0.9765	0.9028	0.8862
PFAS	0.9759	0.9745	0.9026	0.8861
PPAT	0.9647	0.9650	0.9016	0.8851
PAICS	0.9518	0.9509	0.9000	0.8835
ADSL	0.9277	0.9268	0.8961	0.8796
ATIC	0.9036	0.9027	0.8987	0.8822

## Supplementary Materials

The following are available online at https://www.mdpi.com/2218-1989/10/5/184/s1, Table S1: List of genes with a non-empty set of blocked and partially blocked metabolites in Recon 2.2.

## Appendix A. Code Availability

The computer code used in this work to perform all the analyses is available at https://github.com/geryk/Blocked-metabolites-reactions-analysis.

## Appendix B. Datasets Used

All analyses were performed using two genome-scale metabolic network models:

- Recon 2.2: The model is freely available from the Biomodels database, under the identifier MODEL1603150001 [51]. The model is available at http://identifiers.org/biomodels.db/MODEL1603150001.
- An automatically generated metabolic model of the cerebral cortex neuron using the CORDA method [65]. The model is available at https://qutublab.org/apps-code-tools/#Metabolic.

Both models were constrained to uptake only compounds contained within the RPMI_1640 medium for all analyses. (The function constraining the FBA model to RPMI_1640 medium is available at https://github.com/geryk/Blocked-metabolites-reactions-analysis).

## References

[1] Chaaya M., Saab D., Maalouf F.T., Boustany R.M. (2016). Prevalence of Autism Spectrum Disorder in Nurseries in Lebanon: A Cross Sectional Study. J. Autism Dev. Disord..

[2] Pérez-Crespo L., Prats-Uribe A., Tobias A., Duran-Tauleria E., Coronado R., Hervás A., Guxens M. (2019). Temporal and Geographical Variability of Prevalence and Incidence of Autism Spectrum Disorder Diagnoses in Children in Catalonia, Spain. Autism Res..

[3] Baio J. (2014). Prevalence of autism spectrum disorder among children aged 8 years—Autism and developmental disabilities monitoring network, 11 sites, United States, 2010. MMWR Surveill. Summ..

[4] Diallo F.B., Fombonne É., Kisely S., Rochette L., Vasiliadis H.M., Vanasse A., Noiseux M., Pelletier É., Renaud J., St-Laurent D. (2018). Prevalence and Correlates of Autism Spectrum Disorders in Quebec: Prévalence et corrélats des troubles du spectre de l’autisme au Québec. Can. J. Psychiatry.

[5] Barrett B. (2014). Substantial lifelong cost of autism spectrum disorder. J. Pediatr..

[6] Heath A.K., Ganz J.B., Parker R., Burke M., Ninci J. (2015). A Meta-analytic Review of Functional Communication Training Across Mode of Communication, Age, and Disability. Rev. J. Autism Dev. Disord..

[7] Richman D.M., Barnard-Brak L., Grubb L., Bosch A., Abby L. (2015). Meta-analysis of noncontingent reinforcement effects on problem behavior. J. Appl. Behav. Anal..

[8] Kurtz P.F., Chin M.D., Huete J.M., Tarbox R.S.F., O’Connor J.T., Paclawskyj T.R., Rush K.S. (2003). Functional analysus and treatment of self-injurious behavior in young children: A summary of 30 cases. J. Appl. Behav. Anal..

[9] Vissers L.E.L.M., Gilissen C., Veltman J.A. (2016). Genetic studies in intellectual disability and related disorders. Nat. Rev. Genet..

[10] Ramaekers V.T., Sequeira J.M., Blau N., Quadros E.V. (2008). A milk-free diet downregulates folate receptor autoimmunity in cerebral folate deficiency syndrome. Dev. Med. Child. Neurol..

[11] Modabbernia A., Velthorst E., Reichenberg A. (2017). Environmental risk factors for autism: An evidence-based review of systematic reviews and meta-analyses. Mol. Autism.

[12] Rossignol D.A., Genuis S.J., Frye R.E. (2014). Environmental toxicants and autism spectrum disorders: A systematic review. Transl. Psychiatry.

[13] Wang M., Li K., Zhao D., Li L. (2017). The association between maternal use of folic acid supplements during pregnancy and risk of autism spectrum disorders in children: A meta-analysis. Mol. Autism.

[14] Surén P., Roth C., Bresnahan M., Haugen M., Hornig M., Hirtz D., Lie K.K., Lipkin W.I., Magnus P., Reichborn-Kjennerud T. (2013). Association between maternal use of folic acid supplements and risk of autism spectrum disorders in children. JAMA.

[15] Ramaekers V.T., Sequeira J.M., Quadros E.V. (2016). The basis for folinic acid treatment in neuro-psychiatric disorders. Biochimie.

[16] Pu D., Shen Y., Wu J. (2013). Association between MTHFR gene polymorphisms and the risk of autism spectrum disorders: A meta-analysis. Autism Res..

[17] Cario H., Smith D.E.C., Blom H., Blau N., Bode H., Holzmann K., Pannicke U., Hopfner K.P., Rump E.M., Ayric Z. (2011). Dihydrofolate reductase deficiency due to a homozygous DHFR mutation causes megaloblastic anemia and cerebral folate deficiency leading to severe neurologic disease. Am. J. Hum. Genet..

[18] Al-Farsi Y.M., Waly M.I., Deth R.C., Al-Sharbati M.M., Al-Shafaee M., Al-Farsi O., Al-Khaduri M.M., Gupta I., Ali A., Al-Khalili M. (2013). Low folate and vitamin B12 nourishment is common in Omani children with newly diagnosed autism. Nutrition.

[19] Zhao R., Visentin M., Suadicani S.O., Goldman I.D. (2013). Inhibition of the proton-coupled folate transporter (PCFT-SLC46A1) by bicarbonate and other anions. Mol. Pharmacol..

[20] Ramaekers V.T.T., Segers K., Sequeira J.M.M., Koenig M., Van Maldergem L., Bours V., Kornak U., Quadros E.V.V. (2018). Genetic assessment and folate receptor autoantibodies in infantile-onset cerebral folate deficiency (CFD) syndrome. Mol. Genet. Metab..

[21] Frye R.E., Delhey L., Slattery J., Tippett M., Wynne R., Rose S., Kahler S.G., Bennuri S.C., Melnyk S., Sequeira J.M. (2016). Blocking and Binding Folate Receptor Alpha Autoantibodies Identify Novel Autism Spectrum Disorder Subgroups. Front. Neurosci..

[22] Frye R.E., Sequeira J.M., Quadros E.V., James S.J., Rossignol D.A. (2013). Cerebral folate receptor autoantibodies in autism spectrum disorder. Mol. Psychiatry.

[23] Nagarajan R.P., Hogart A.R., Gwye Y., Martin M.R., LaSalle J.M. (2006). Reduced MeCP2 expression is frequent in autism frontal cortex and correlates with aberrant MECP2 promoter methylation. Epigenetics.

[24] Alex A.M., Saradalekshmi K.R., Shilen N., Suresh P.A., Banerjee M. (2019). Genetic association of DNMT variants can play a critical role in defining the methylation patterns in autism. IUBMB Life.

[25] Sanders S.J., He X., Willsey A.J., Ercan-Sencicek A.G., Samocha K.E., Cicek A.E., Murtha M.T., Bal V.H., Bishop S.L., Dong S. (2015). Insights into Autism Spectrum Disorder Genomic Architecture and Biology from 71 Risk Loci. Neuron.

[26] Yi P., Melnyk S., Pogribna M., Pogribny I.P., Hine R.J., James S.J. (2000). Increase in plasma homocysteine associated with parallel increases in plasma S-adenosylhomocysteine and lymphocyte DNA hypomethylation. J. Biol. Chem..

[27] James S.J., Cutler P., Melnyk S., Jernigan S., Janak L., Gaylor D.W., Neubrander J.A. (2004). Metabolic biomarkers of increased oxidative stress and impaired methylation capacity in children with autism. Am. J. Clin. Nutr..

[28] Schaevitz L.R., Berger-Sweeney J.E. (2012). Gene-environment interactions and epigenetic pathways in autism: The importance of one-carbon metabolism. ILAR J..

[29] Hamlin J.C., Pauly M., Melnyk S., Pavliv O., Starrett W., Crook T.A., James S.J. (2013). Dietary intake and plasma levels of choline and betaine in children with autism spectrum disorders. Autism Res. Treat..

[30] Tanaka M., Sato A., Kasai S., Hagino Y., Kotajima-Murakami H., Kashii H., Takamatsu Y., Nishito Y., Inagaki M., Mizuguchi M. (2018). Brain hyperserotonemia causes autism-relevant social deficits in mice. Mol. Autism.

[31] Rossignol D.A., Frye R.E. (2011). Melatonin in autism spectrum disorders: A systematic review and meta-analysis. Dev. Med. Child. Neurol..

[32] Esmaiel N.N., Ashaat E.A., Mosaad R., Fayez A., Ibrahim M., Abdallah Z.Y., Issa M.Y., Salem S., Ramadan A., El Wakeel M.A. (2020). The potential impact of COMT gene variants on dopamine regulation and phenotypic traits of ASD patients. Behav. Brain Res..

[33] Frye R.E., DeLatorre R., Taylor H.B., Slattery J., Melnyk S., Chowdhury N., James S.J. (2013). Metabolic effects of sapropterin treatment in autism spectrum disorder: A preliminary study. Transl. Psychiatry.

[34] Naviaux R.K. (2018). Antipurinergic therapy for autism-An in-depth review. Mitochondrion.

[35] Naviaux J.C., Wang L., Li K., Bright A., Alaynick W.A., Williams K.R., Powell S.B., Naviaux R.K. (2015). Antipurinergic therapy corrects the autism-like features in the Fragile X (Fmr1 knockout) mouse model. Mol. Autism.

[36] Naviaux J.C., Schuchbauer M.A., Li K., Wang L., Risbrough V.B., Powell S.B., Naviaux R.K. (2014). Reversal of autism-like behaviors and metabolism in adult mice with single-dose antipurinergic therapy. Transl. Psychiatry.

[37] Howsmon D.P., Kruger U., Melnyk S., James S.J., Hahn J. (2017). Classification and adaptive behavior prediction of children with autism spectrum disorder based upon multivariate data analysis of markers of oxidative stress and DNA methylation. PLoS Comput. Biol..

[38] Li G., Lee O., Rabitz H. (2018). High efficiency classification of children with autism spectrum disorder. PLoS ONE.

[39] Hollowood K., Melnyk S., Pavliv O., Evans T., Sides A., Schmidt R.J., Hertz-Picciotto I., Elms W., Guerrero E., Kruger U. (2018). Maternal metabolic profile predicts high or low risk of an autism pregnancy outcome. Res. Autism Spectr. Disord..

[40] Vargason T., Kruger U., Roth E., Delhey L.M., Tippett M., Rose S., Bennuri S.C., Slattery J.C., Melnyk S., James S.J. (2018). Comparison of Three Clinical Trial Treatments for Autism Spectrum Disorder Through Multivariate Analysis of Changes in Metabolic Profiles and Adaptive Behavior. Front. Cell. Neurosci..

[41] Vargason T., Howsmon D.P., Melnyk S., James S.J., Hahn J. (2017). Mathematical modeling of the methionine cycle and transsulfuration pathway in individuals with autism spectrum disorder. J. Theor. Biol..

[42] Raman K., Chandra N. (2009). Flux balance analysis of biological systems: Applications and challenges. Brief. Bioinform..

[43] Edwards J.S., Covert M., Palsson B. (2002). Metabolic modelling of microbes: The flux-balance approach. Environ. Microbiol..

[44] Lee J.M., Gianchandani E.P., Papin J.A. (2006). Flux balance analysis in the era of metabolomics. Brief. Bioinform..

[45] Drozdov-Tikhomirov L.N., Scurida G.I., Davidov A.V., Alexandrov A.A., Zvyagilskaya R.A. (2006). Mathematical modeling of living cell metabolism using the method of steady-state stoichiometric flux balance. J. Bioinform. Comput. Biol..

[46] Sahoo S., Franzson L., Jonsson J.J., Thiele I. (2012). A compendium of inborn errors of metabolism mapped onto the human metabolic network. Mol. Biosyst..

[47] Karlstädt A., Fliegner D., Kararigas G., Ruderisch H.S., Regitz-Zagrosek V., Holzhütter H.G. (2012). CardioNet: A human metabolic network suited for the study of cardiomyocyte metabolism. BMC Syst. Biol..

[48] Gudmundsson S., Thiele I. (2010). Computationally efficient flux variability analysis. BMC Bioinform..

[49] Massaiu I., Pasotti L., Sonnenschein N., Rama E., Cavaletti M., Magni P., Calvio C., Herrgård M.J. (2019). Integration of enzymatic data in Bacillus subtilis genome-scale metabolic model improves phenotype predictions and enables in silico design of poly-γ-glutamic acid production strains. Microb. Cell Fact..

[50] Krsička D., Geryk J., Vlčková M., Havlovicová M., Macek M., Pourová R. (2017). Identification of likely associations between cerebral folate deficiency and complex genetic- and metabolic pathogenesis of autism spectrum disorders by utilization of a pilot interaction modeling approach. Autism Res..

[51] Swainston N., Smallbone K., Hefzi H., Dobson P.D., Brewer J., Hanscho M., Zielinski D.C., Ang K.S., Gardiner N.J., Gutierrez J.M. (2016). Recon 2.2: From reconstruction to model of human metabolism. Metabolomics.

[52] Smart A.G., Amaral L.A.N., Ottino J.M. (2008). Cascading failure and robustness in metabolic networks. Proc. Natl. Acad. Sci. USA.

[53] Zikanova M., Skopova V., Hnizda A., Krijt J., Kmoch S. (2010). Biochemical and structural analysis of 14 mutant ADSL enzyme complexes and correlation to phenotypic heterogeneity of adenylosuccinate lyase deficiency. Hum. Mutat..

[54] Marie S., Heron B., Bitoun P., Timmerman T., Van Den Berghe G., Vincent M.F. (2004). AICA-ribosiduria: A novel, neurologically devastating inborn error of purine biosynthesis caused by mutation of ATIC. Am. J. Hum. Genet..

[55] Blau N., Ichinose H., Nagatsu T., Heizmann C.W., Zacchello F., Burlina A.B. (1995). A missense mutation in a patient with guanosine triphosphate cyclohydrolase I deficiency missed in the newborn screening program. J. Pediatr..

[56] Chien Y.H., Chiang S.C., Huang A., Lin J.M., Chiu Y.N., Chou S.P., Chu S.Y., Wang T.R., Hwu W.L. (2001). Treatment and outcome of Taiwanese patients with 6-pyruvoyltetrahydropterin synthase gene mutations. J. Inherit. Metab. Dis..

[57] Verbeek M.M., Willemsen M.A.A.P., Wevers R.A., Lagerwerf A.J., Abeling N.G.G.M., Blau N., Thöny B., Vargiami E., Zafeiriou D.I. (2008). Two Greek siblings with sepiapterin reductase deficiency. Mol. Genet. Metab..

[58] Keil K.P., Lein P.J. (2016). DNA methylation: A mechanism linking environmental chemical exposures to risk of autism spectrum disorders?. Environ. Epigenetics.

[59] Melnyk S., Fuchs G.J., Schulz E., Lopez M., Kahler S.G., Fussell J.J., Bellando J., Pavliv O., Rose S., Seidel L. (2012). Metabolic imbalance associated with methylation dysregulation and oxidative damage in children with autism. J. Autism Dev. Disord..

[60] Jurecka A., Zikanova M., Kmoch S., Tylki-Szymańska A. (2015). Adenylosuccinate lyase deficiency. J. Inherit. Metab. Dis..

[61] Chung B.K.S., Lee D.Y. (2009). Flux-sum analysis: A metabolite-centric approach for understanding the metabolic network. BMC Syst. Biol..

[62] Maddocks O.D.K., Labuschagne C.F., Adams P.D., Vousden K.H. (2016). Serine Metabolism Supports the Methionine Cycle and DNA/RNA Methylation through De Novo ATP Synthesis in Cancer Cells. Mol. Cell.

[63] Schellenberger J., Que R., Fleming R.M.T., Thiele I., Orth J.D., Feist A.M., Zielinski D.C., Bordbar A., Lewis N.E., Rahmanian S. (2011). Quantitative prediction of cellular metabolism with constraint-based models: The COBRA Toolbox v2.0. Nat. Protoc..

[64] Krsička D., Vlčková M., Havlovicová M. (2014). The Significance of Cerebral Folate Deficiency for the Development and Treatment of Autism Spectrum Disorders. Int. J. Biomed. Healthc..

[65] Schultz A., Qutub A.A. (2016). Reconstruction of Tissue-Specific Metabolic Networks Using CORDA. PLoS Comput. Biol..

